# Attempts to Achieve Targeted Covalent Inhibition of Hsp90*β*

**DOI:** 10.1111/cbdd.70290

**Published:** 2026-04-12

**Authors:** Terin D'Amico, Tyelor S. Reynolds, Michael A. Serwetnyk, Brian S. J. Blagg

**Affiliations:** ^1^ Department of Chemistry and Biochemistry, Warren Center for Drug Discovery The University of Notre Dame Notre Dame Indiana USA

**Keywords:** acrylamides, Fluorosulfonyl, Hsp90, Hsp90*β*, isoform‐selective inhibition, lysine, Michael acceptors, targeted covalent inhibition

## Abstract

Recent advances in targeted covalent inhibition have broadened the therapeutic landscape via the development of warheads that target various nucleophilic amino acids. Although cysteine was once the primary focus of such efforts, lysine has emerged as another appealing residue for covalent modification, due to its high prevalence within the proteome, functional importance, and low mutation rate. Despite this amino acid's reduced nucleophilicity at physiological pH, rational, structure‐based techniques have enabled the identification of inhibitors that exploit the unique protein microenvironment that a given lysine occupies. Strategies to inhibit Hsp90 via covalent modification of Lys58 include the use of *N*‐acyl‐*N*‐aryl sulfonamide (ArNASA) warheads and sulfur(VI) fluoride exchange (SuFEx) chemistry, both of which have demonstrated enhanced cellular selectivity and potency. However, such compounds fail to distinguish between the > 95% identical nature of the ligand binding sites in cytosolic Hsp90*α* and Hsp90*β*. In this study, the incorporation of electrophilic warheads onto Hsp90*β*‐selective inhibitors resulted in compounds that demonstrate favorable binding to Hsp90*β*, yet subsequent analyses to confirm covalent modification of Lys58 were inconclusive. Nevertheless, the kinetics of inhibitor binding to the Hsp90 N‐terminal ATP‐binding pocket were obtained, whereby some compounds exhibited their highest affinity towards Hsp90*β* after a 2 h incubation.

## Graphical abstract

Electrophilic warheads were incorporated into Hsp90*β*‐selective inhibitors to covalently modify Lys58, which occupies the solvent‐exposed region of the binding pocket. Although many inhibitors display nanomolar affinity towards Hsp90*β* over a 24‐h period, analyses to determine covalent bond formation with this residue were inconclusive. Interestingly though, several compounds exhibited their highest affinity towards the protein after a 2‐h incubation, which reveals insights into the binding kinetics of inhibitors that occupy the Hsp90 N‐terminal pocket.

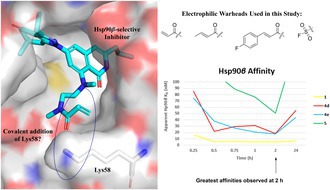

**Abbreviations**
ACN: acetonitrile
ArNASA: *N*‐acyl‐*N*‐aryl sulfonamide
ATP: adenosine triphosphate
DCM: dichloromethane
DIPEA: *N N*‐diisopropylethylamine
DMSO: dimethylsulfoxide
FP: fluorescence polarization
HCl: hydrochloric acid
Hsp90: 90‐kDa heat shock protein
Lys58: lysine 58
MeOH: methanol
NASA: *N*‐acyl‐*N*‐alkyl sulfonamide
PPIs: protein–protein interactions
SuFEx: sulfur (VI) fluoride exchange
TCI: targeted covalent inhibitor
TEA: triethylamine

## 1 Introduction

Recent advances in targeted covalent inhibitors (TCIs) have reinvigorated interest in irreversible enzyme modulation (Gehringer 2020; Singh et al. 2011; Baillie 2016). In contrast to traditional small molecule inhibitors, which rely upon multiple non‐covalent interactions to facilitate target engagement, TCIs selectively and irreversibly bind proteins via the formation of a covalent bond between an electrophilic warhead and a proximal nucleophile. Consequently, enhanced potency and prolonged residence time are notable advantages of TCIs. Initial covalent inhibitors achieved these effects by exploiting the high reactivity of cysteine thiols via acrylamides or other related *α*,*β*‐unsaturated Michael acceptors (Pace and Weerapana 2013). However, the field of TCI development has evolved such that cysteine‐targeting warheads now operate under a variety of mechanisms of action: alkenyl‐ or alkynyl‐substituted heterocycles for analogous nucleophilic additions, *α*‐substitution with halogens or other leaving groups for S_N_2 displacements, appropriately substituted (hetero)arenes for S_N_Ar reactions, bicyclo[1.1.0]butanes as strain release agents, as well as nitriles, ketones, and aldehydes for reversible additions (Hillebrand et al. 2024; McAulay et al. 2020; Seki et al. 2021; Gehringer and Laufer 2019; Reddi et al. 2023; Zambaldo et al. 2020; Gianatassio et al. 2016; Lopchuk et al. 2017; Bonatto et al. 2022). For proteins that lack this residue at or near the ligand‐binding site, these same chemical principles have been applied towards the design of alternative warheads that target other nucleophilic species (Hillebrand et al. 2024; Huang and Jones 2023; Chalker et al. 2009; Shannon and Weerapana 2015; Mukherjee and Grimster 2018; Pettinger et al. 2017; Cuesta and Taunton 2019). Such efforts have led to the successful modification of amino acids including arginine, methionine, histidine, and proline. However, serine/threonine, glutamic/aspartic acid, tyrosine, and lysine have become the more notable candidates for TCIs (Gehringer and Laufer 2019; Zhang et al. 2022; Crichlow et al. 2009).

Lysine has emerged as another key amino acid for the development of TCIs, largely due to its greater abundance within the human proteome over cysteine (5.9% vs. 1.9%) (Echols et al. 2002; Matos et al. 2018; Rosen and Francis 2017; Boll and Raines 2022), which broadens the range of druggable proteins. In fact, a recent analysis conducted by Abbasov and colleagues utilized a small library of aminophilic molecules to identify > 800 chemically modifiable lysines across 581 unique proteins (Abbasov et al. 2021). Although the authors concluded that lysine modulation is a rare phenomenon (13,785 lysines on 3552 unique proteins in total were initially quantified), they maintained that these values are not representative of the entire human proteome (Abbasov et al. 2021). Even among the small fraction of structurally and functionally diverse proteins that were identified, many of the lysines were located within enzyme active sites or known to participate in protein–protein or protein–RNA interactions, which indicates that TCIs for this amino acid can act via multiple mechanisms of action (Abbasov et al. 2021). Furthermore, lysine is integral to various catalytic processes and plays a vital role in maintaining the structural integrity and regulatory function of proteins via post‐translational modification, which reduces the likelihood of viable mutations. Altogether, these attributes have made lysine an attractive residue for the development of TCIs in recent years.

However, there are significant issues associated with lysine targeting. For instance, lysine is typically less nucleophilic than cysteine and is further complicated by the fact that lysine is primarily protonated at physiological pH (~99.9%; pKa ≈10.5) (Rosen and Francis 2017; Platzer et al. 2014). Nevertheless, the specific protein microenvironment for a given lysine can influence its reactivity via alterations to the pKa (Isom et al. 2011). Secondly, the *ε*‐amine of lysine resides at the end of a long, flexible alkyl chain, which means that TCIs that engage this amino acid are entropically disfavored. Lastly, the relatively high abundance of lysine, as compared to other nucleophilic residues (Rosen and Francis 2017), increases the potential for off‐target effects. Consequently, the design of TCIs must involve rational, structure‐based methods to exploit the unique microenvironment of the active site, establish a pharmacophore, and enhance selectivity. Advanced techniques, such as computational modeling and high‐resolution crystallography, are available for evaluating the lysine's role within the active site. Such methods also enable opportunities for the optimization of non‐covalent scaffolds via warhead screenings and have led to the adoption of an “electrophile approach” towards drug design in recent years (You et al. 2025).

These strategies have been utilized to develop TCIs of the 90‐kDa heat shock protein (Hsp90). This ubiquitous and highly conserved molecular chaperone is essential for the maintenance of cellular proteostasis and the biologically active conformations of > 400 client protein substrates (Taipale et al. 2012). Because many of these clients are associated with the 10 hallmarks of cancer, Hsp90 inhibition simultaneously impacts multiple oncogenic pathways via a single target, yet manifests outcomes similar to combination therapy (Hanahan and Weinberg 2000, 2011; Blagg and Kerr 2006; Vartholomaiou et al. 2016; Whitesell and Lindquist 2005). Proteomic studies that identified Hsp90 as a target have typically done so via chemical probes that modulate either cysteines or tyrosines, which supports the design of TCIs with moieties that modulate these amino acids (Fu et al. 2025). Additionally, Celastrol A, sulforaphane, Withaferin A, and Kongensin A are natural products that allosterically disrupt protein–protein interactions (PPIs) between Hsp90 and its various co‐chaperones via the formation of covalent bonds with cysteine residues (Grover et al. 2011; Sreeramulu et al. 2009; Zhang et al. 2008, 2009; Yu et al. 2010; Li et al. 2011, 2012, 2016). Furthermore, several other natural products and small molecules have been discovered or developed to modulate the cytosolic Hsp90*α* and Hsp90*β* isoforms, though these efforts have also mostly targeted cysteine residues (Abiko et al. 2017; Martínez‐Ruiz et al. 2005; Dai et al. 2019, 2020; Li et al. 2021; Song et al. 2015; Zhang et al. 2014, 2020, 2011).

The design of TCIs that target Hsp90's Lys58 involves several challenges, as evidenced by a lone study that found engagement with this residue via an *ortho*‐phthalaldehyde (OPA) S‐S‐alkyne probe whose warhead serves as a Paal‐Knorr agent (Wang et al. 2024, 2021). Lys58's location at the solvent‐exposed region of the Hsp90 N‐terminal ATP‐binding pocket requires inhibitors to balance high binding affinity and selective reactivity. In 2018, Tamura and colleagues identified an *N*‐acyl‐*N*‐alkyl sulfonamide (NASA) warhead, comprised of a phenylsulfonamide and an electron‐withdrawing cyanomethyl group, that could modify Lys58 when appended to the Hsp90 inhibitor, PU‐H71 (Tamura et al. 2018). However, activity of this moiety is compromised by its sensitivity to water, as well as the other proteins present in cell culture serum/media (Tamura et al. 2018; Tamura and Hamachi 2024; Kawano et al. 2023). A subsequent optimization study on these NASAs resulted in 2nd generation, lysine‐reactive *N*‐acyl‐*N*‐aryl sulfonamide (ArNASA) warheads, which are more stable and exhibit improved activity and selectivity towards this residue (Tamura and Hamachi 2024; Kawano et al. 2023). In fact, these warheads were found to overcome difficulties associated with resistance mutations in Bruton's tyrosine kinase (BTK), as well as display increased effectiveness against Hsp90 (Kawano et al. 2023). Meanwhile, in 2020, Cuesta and coworkers attached an aryl sulfonyl fluoride warhead onto PU‐H71, which enantioselectively promoted covalent bond formation with Lys58 via proximity‐induced rate acceleration (Cuesta et al. 2020). Unfortunately, these inhibitors are not isoform‐selective, as they target both Hsp90*α* and Hsp90*β*, which share > 95% identity within the ATP‐binding site (Tamura et al. 2018; Kawano et al. 2023; Cuesta et al. 2020). Hence, overcoming these challenges requires a structure‐based approach to optimize interactions between the inhibitor and the ATP‐binding site, while engaging Lys58 at the protein‐solution interface. Fortunately, our lab has succeeded in exploiting the subtle differences within the Hsp90 N‐terminal ATP‐binding pockets to develop selective inhibitors of all four Hsp90 isoforms (Khandelwal et al. 2017; Khandelwal et al. 2018; Xu et al. 2025; Pugh et al. 2024; Serwetnyk et al. 2025; Que et al. 2018; Mishra, Liu, et al. 2021; Mishra et al. 2016; Mishra, Khandelwal, et al. 2021; Mishra et al. 2022; Chaudhury et al. 2021; Merfeld et al. 2023; D'Amico et al. 2025; Crowley et al. 2017). For instance, the ATP‐binding pocket within Hsp90*α* possesses Ser52 and Ile91, whereas the Hsp90*β* pocket contains Ala52 and Leu91 at these respective positions (Khandelwal et al. 2018; Mishra, Khandelwal, et al. 2021). In recent years, our lab has designed a series of phenols that achieve Hsp90*α* selectivity by leveraging the subtleties in the water‐mediated hydrogen bonding network at the bottom of the pocket created by the Ser/Ala52 difference (Mishra, Khandelwal, et al. 2021; Mishra et al. 2022). Meanwhile, our isoquinolinone‐containing scaffold's Hsp90*β* selectivity arises from the Ile/Leu91 difference, since a “methyl migration” between these residues creates a small, hydrophobic sub‐pocket that is exclusive to this isoform and occupied by these inhibitors (Serwetnyk et al. 2025; Mishra, Liu, et al. 2021; D'Amico et al. 2025). Encouragingly, many of these compounds, such as our Hsp90*β*‐selective inhibitors, are amenable to the incorporation of electrophilic moieties. Sulfur (VI) fluoride exchange (SuFEx) has also emerged as a valuable reagent for the development of covalent inhibitors by offering a robust and versatile approach for chemical synthesis (Huang and Jones 2023; Dong et al. 2014; Hansen et al. 2025). The utility of SuFEx is highlighted by its ability to form stable sulfur‐containing bonds with various nucleophiles, enabling the modification of functional groups with high specificity and potency.

Therefore, the combination of Hsp90 isoform‐selective inhibitors with electrophilic warheads that covalently target Lys58 offers a promising approach for cancer therapy by disrupting Hsp90's ATPase activity and providing sustained inhibition through covalent modification. Herein, we present the development and biological characterization of proposed Hsp90*β*‐selective covalent inhibitors.

## 2 Materials and Methods

### 2.1 Chemistry

#### 2.1.1 General Information

All reagents and solvents were purchased from commercially available sources. All reactions were performed in oven‐ or flame‐dried glassware under an argon atmosphere, unless otherwise stated. Flash column chromatography was performed using silica gel (40–63 *μ*m particle size). A Bruker 400 MHz or 500 MHz spectrometer was used for ^1^H‐, ^13^C‐, and ^19^F‐NMR spectra acquisition. Coupling constants (*J*) are reported in Hertz, chemical shifts are referenced to the residual deuterated solvent peak, and peak *δ* values are reported in parts per million (ppm). High‐resolution mass spectral data were obtained on a time‐of‐flight mass spectrometer, and analysis was performed using electrospray ionization. Thin‐layer chromatography was performed with TLC silica gel 60F254 plates purchased from Millipore Sigma and visualized by UV light (254 nm). All materials were handled in accordance with their material safety data sheets (MSDS), and so no unexpected or unusually high safety hazards were encountered with the methods and conditions presented in this study.

#### 2.1.2 Synthesis of Compound 1

Preparation of non‐covalent control compound **1** was accomplished via the synthetic route described in prior studies (Mishra, Liu, et al. 2021; D'Amico et al. 2025). Characterization of this species can be found in the report published by D'Amico et al. (2025).

#### 2.1.3 Synthesis of Intermediate *2*

Preparation of late‐stage aryl fluoride **2** was accomplished via the synthetic route described in prior studies from our lab (Serwetnyk et al. 2025; Mishra, Liu, et al. 2021). Characterization of this species can be found in the report published by Serwetnyk et al. (2025).

#### 2.1.4 General Synthesis of Intermediates *3a–c*

DIPEA (3.79 mmol, 3 equiv.) was added to a pressure tube charged with aryl fluoride intermediate **2** (1.26 mmol, 1 equiv.) and the appropriate amine (3.79 mmol, 3 equiv.) dissolved in DMSO (12 mL). **Note:** For the preparation of **3a**, this reaction required 10 equivalents (12.6 mmol) of the ethylenediamine to ensure that enough was present in the solution and not trapped in the headspace of the reaction tube. The vessel was sealed, and the reaction was stirred at 140°C for 12 h. Upon cooling to room temperature, water (50 mL) was added to the solution, and the aqueous layer was extracted with ethyl acetate (3 × 50 mL). The combined organic fractions were washed with water (5 × 50 mL), then with brine (50 mL), dried over sodium sulfate, filtered, and concentrated in vacuo. The residue was purified via column chromatography (5% MeOH in DCM) and then further purified via preparatory TLC (3% MeOH in DCM) to give the products as light‐yellow solids.

#### 2.1.5 Synthesis of Intermediate *3d*

A 4.0 N solution of HCl in 1,4‐dioxane (1.36 mL, 5.45 mmol, 5 equiv.) was added dropwise to a solution of intermediate **3c** (628 mg, 1.09 mmol, 1 equiv.) in DCM (10 mL) at 0°C. The reaction was stirred at room temperature for 20 h, after which the solvents were removed in vacuo. The residue was partitioned between water (10 mL) and ethyl acetate (10 mL), and the aqueous layer was extracted with ethyl acetate (3 × 10 mL). The combined organic fractions were washed with brine (10 mL), dried over sodium sulfate, filtered, and concentrated in vacuo. The residue was purified via column chromatography (0%–20% NH_3_ in MeOH solution in DCM) and then further purified via recrystallization in ethyl acetate and hexanes to provide **3d** as an off‐white amorphous solid.

#### 2.1.6 General Synthesis of Compounds *4a–i*

The appropriate acid chloride (2 equiv.) was added dropwise to a solution of amine **3a**, **3b**, or **3d** (100 mg, 1 equiv.) and TEA (2 equiv.) in DCM (4.0 mL) at 0°C. The reaction was subsequently stirred at room temperature until the consumption of starting material was observed, as determined via TLC (~2 h). The reaction was quenched with MeOH (1.0 mL), and the organic layer was washed with water (2.0 mL), then with brine (2.0 mL), dried over sodium sulfate, filtered, and concentrated *in vacuo*. The residue was purified via column chromatography (5% MeOH in DCM) and then further purified via preparatory TLC (3% MeOH in DCM) to give the products as colorless or light‐yellow solids.

#### 2.1.7 Synthesis of Compound *5*

Preparation of this compound was adapted from the procedures reported by Guo et al. (2017). Intermediate **3b** (50 mg, 0.11 mmol, 1 equiv.) and 1‐(fluorosulfonyl)‐2,3‐dimethyl‐1*H*‐imidazol‐3‐ium trifluoromethanesulfonate (46 mg, 0.14 mmol, 1.3 equiv.) were dissolved in acetonitrile (2.0 mL), and the reaction was stirred at room temperature until the complete consumption of starting material was observed, as determined via TLC (~2 h). The reaction was quenched with water (2.0 mL), and the aqueous layer was extracted with ethyl acetate (3 × 2.0 mL). The combined organic fractions were washed with water (4.0 mL), then with brine (4.0 mL), dried over sodium sulfate, filtered, and concentrated in vacuo to give compound **5** as a colorless solid.

### 2.2 Procedure for the Fluorescence Polarization (FP) Assay

The FP assay was performed in 96‐well format in black, flat‐bottom plates (Santa Cruz Biotechnology) with a final volume of 100 *μ*L. Twenty‐five microliters of assay buffer (20 mM HEPES, 50 mM KCl, 10.5 mM MgCl_2_, 20 mM Na_2_MoO_4_, 0.01% NP‐40 detergent (NP‐40), and pH 7.3 with fresh 2 mM dithiothreitol (DTT) and 0.1 mg mL^−1^ bovine *γ*‐globulin (BGG) added before use), 25 *μ*L of assay buffer containing 6 nM FITC‐GDA (fluorescent tracer, stock in DMSO and diluted in assay buffer) and 50 *μ*L of assay buffer containing 10 nM of Hsp90*α* and Hsp90*β* were added to each well. Compounds were tested in triplicate wells (1% DMSO final concentration). For each plate, wells containing buffer only (background), tracer in buffer only (low polarization control), and protein and tracer in buffer with 1% DMSO (high polarization control) were included. Plates were incubated at 4°C with rocking for 24 h. Polarization values (in mP units) were measured at 37°C with an excitation filter at 485 nm and an emission filter at 528 nm. Polarization values were correlated to % tracer bound and compound concentrations. The concentration at which the tracer was 50% displaced by the compound of interest (IC_50_) was obtained using Microsoft Excel and reported as an average and standard deviation from triplicate runs.

For the time‐dependent FP assay, the single dose concentrations were selected to be twice the apparent *K*_*D*_ obtained at 2 h. Time‐dependent assay plates were incubated in the plate reader at 37°C for up to 4 h between measurements and then at 4°C overnight prior to the 24 h reading.

### 2.3 General Cell Culture Procedures

A549, MDA‐MB‐231, and SKOV‐3 cells were grown in a water‐jacketed incubator at 37°C with 5% CO_2_. A549, MDA‐MB‐231, and SKOV‐3 cells were cultured in RPMI‐1640, DMEM, and McCoy's media, respectively, all of which were supplemented with 10% HI FBS (Gibco, 10,438‐026) and 1% Pen‐Strep (VWR, K952‐100 mL).

### 2.4 MTS Assay Procedure

A549, MDA‐MB‐231, and SKOV‐3 cells were grown to confluence, and then seeded in clear, flat‐bottomed 96‐well plates at a density of 1000 cells per well. After an overnight attachment period, cells were treated either with 0.5% DMSO (vehicle control) or compound. The number of viable cells was determined at 72 h using the CellTiter 96 Aqueous One Solution Cell Proliferation assay kit (Promega Corp., Madison, WI), in accordance with the manufacturer's instructions. 20 *μ*L of CellTiter 96 solution was added to each well, and the plates were incubated for 3.25 h at 37°C, after which the absorbance of each well was measured at a wavelength of 490 nm with a microplate spectrophotometer (Biotek Epoch). All assay runs were performed in triplicate, and the antiproliferative values for each compound were determined from 3 independent experiments. Cell growth of treated plates was calculated relative to an untreated time zero plate measured at the time of treatment. Growth inhibition curves, half‐maximal inhibitory concentrations (IC_50_), and statistical analyses were calculated with GraphPad Prism V9.

### 2.5 Procedure for Molecular Modeling and Docking Studies

**FIGURE 1:**
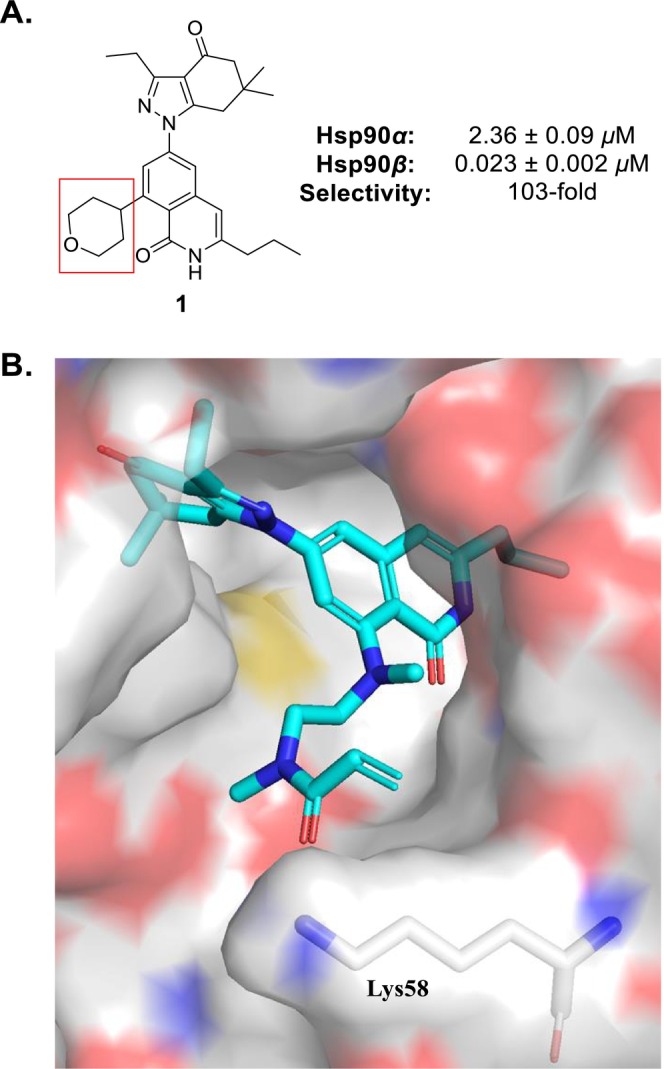
Rationale for the development of Hsp90*β*‐selective inhibitors with electrophilic warheads. (A) Compound **1** is a non‐covalent Hsp90*β*‐selective inhibitor that is used as a control for these studies. Apparent *K*_*D*_ values were determined via a fluorescence polarization (FP) assay. The tetrahydropyran ring of **1** (boxed in red) occupies the protein‐solvent interface of the Hsp90 ATP‐binding pocket. (B) Molecular docking studies indicate that proposed covalent Hsp90*β*‐selective inhibitors (such as **4a**) occupy the Hsp90*β* ATP‐binding pocket (PDB: 1UYM (Wright et al. 2004)) in a similar manner as earlier compounds, while the acrylamide warhead lies near Lys58.

The Maestro program from Schrödinger was used for molecular modeling and docking studies. The co‐crystal structure of PU‐3 bound to Hsp90*β* (PDB: 1UYM (Wright et al. 2004)) was utilized for modeling experiments. PyMOL was used for further visualization and in the preparation of Figure 1B.

## 3 Results and Discussion

### 3.1 Design and Synthesis of Hsp90*β*‐Selective Inhibitors With Electrophilic Warheads

Lys58 is a “gate‐way residue” located within the solvent‐exposed region of the Hsp90 N‐terminal ATP‐binding pocket (Mishra, Liu, et al. 2021). Based on this observation, the appendages of Hsp90*β*‐selective inhibitors (such as the tetrahydropyran ring of **1**, Figure 1A, boxed in red) that occupy this region of the pocket feature polar moieties to facilitate hydrogen bonding (Mishra, Liu, et al. 2021). However, the optimal distance to engage Lys58, which is crucial for covalent bond formation, remains unknown. Therefore, the design strategy for Lys58‐targeting Hsp90*β*‐selective inhibitors was to attach electrophilic warheads onto a previously disclosed scaffold via amines of varying length and/or flexibility. Molecular docking studies were conducted on proposed covalent Hsp90*β*‐selective inhibitors within the Hsp90*β* ATP‐binding pocket (PDB: 1UYM (Wright et al. 2004)) (Figure 1B). According to this investigation, the scaffold occupies the pocket in a similar manner as prior compounds (Mishra, Liu, et al. 2021), while the acrylamide warhead extends towards Lys58. These preliminary results suggest that the incorporation of such electrophilic groups onto our Hsp90*β*‐selective inhibitors can achieve a balance between target affinity, selectivity, and residence time. To this end, an ethylenediamine, a piperazine ring, and a 4‐aminopiperidine were chosen as amine linkers. Meanwhile, the initial selection of covalent warheads included an assortment of commonly utilized acrylamides to assess the preferred ligand length. Although acrylamides are commonly used to target cysteine residues, we hypothesized that their electrophilicity will be sufficient to react with Lys58. Consequently, whichever amine tether results in the highest affinity towards Hsp90*β* will be the one onto which the fluorosulfonyl warhead that Cuesta and colleagues previously used to modulate Lys58 is installed (Cuesta et al. 2020).

**SCHEME 1:**
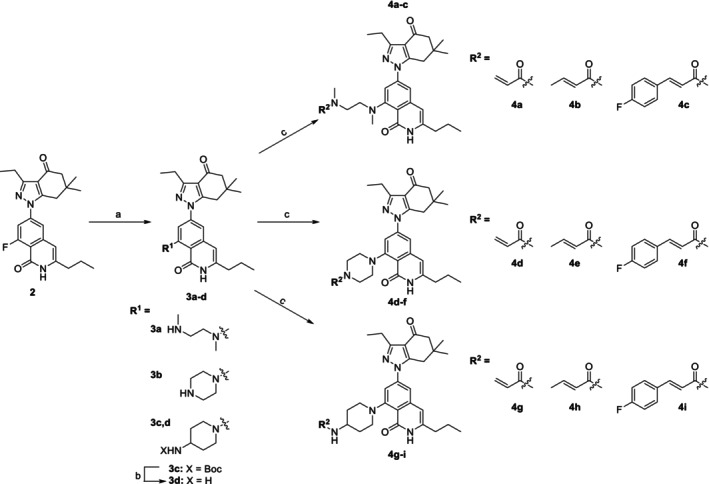
Synthesis of Hsp90*β*‐selective inhibitors with acrylamide warheads. *Reagents and conditions*: (a) Amine, DIPEA, DMSO, 140°C, 12 h, 68%–86%; (b) HCl (4.0 M in dioxane), DCM, 0°C to rt., 20 h, 77%; (c) Acid chloride, TEA, DCM, 0°C to rt., 2 h, 5%–43%.

**SCHEME 2:**
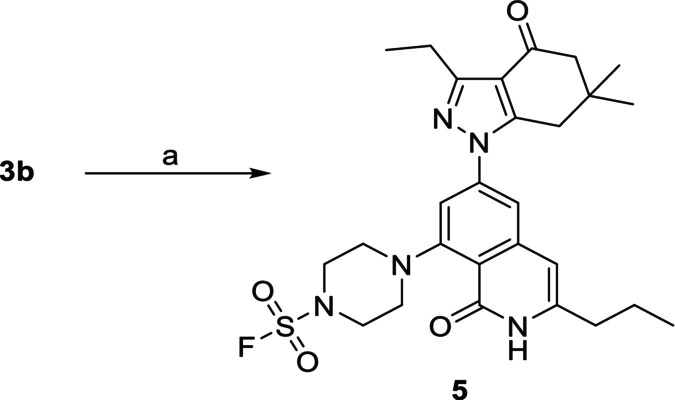
Synthesis of Hsp90*β*‐selective inhibitor with a fluorosulfonyl warhead. *Reagents and conditions*: (a) Fluorosulfuryl imidazolium triflate, TEA, ACN, rt., 2 h, 20%.

Synthesis of Hsp90*β*‐selective inhibitors with electrophilic warheads is depicted in Scheme 1. Aryl fluoride **2**, which was prepared according to an established route (Serwetnyk et al. 2025; Mishra, Liu, et al. 2021; D'Amico et al. 2025), underwent a S_N_Ar reaction with various amines to provide intermediates, **3a–c**. For intermediate **3c**, 4‐(*N*‐Boc‐amino)piperidine was utilized to ensure addition at the secondary amine, and subsequent exposure to acidic conditions cleaved the carbamate protecting group to generate **3d**. Successive treatment of **3a**, **3b**, and **3d** with an appropriate acid chloride resulted in compounds **4a–i**, which feature acrylamides to determine the optimal length needed to engage Lys58 via Michael addition. However, bioorthogonal reactions, such as sulfur (VI) fluoride exchange (SuFEx), were also proposed to enable covalent bond formation between our inhibitors and this residue (Cuesta et al. 2020). Consequently, the reaction between **3b** and a fluorosulfuryl imidazolium triflate salt was performed to furnish compound **5**, which bears a more amenable handle for SuFEx chemistry (Scheme 2) (Guo et al. 2017).

### 3.2 Evaluation of Hsp90 Binding Affinity

Prior to the attachment of covalent warheads, intermediates **3a**, **3b**, and **3d** underwent evaluation via a standard fluorescence polarization (FP) assay to assess whether the amine tethers maintain affinity/selectivity for Hsp90*β* (Table 1). Compound **1** has previously been found to bind Hsp90*α* and Hsp90*β* with apparent *K*_*D*_ values of 2.36 and 0.023 *μ*M, respectively, which corresponds to 103‐fold selectivity towards the desired isoform. As discussed elsewhere, oxygen‐containing heterocycles appear to be most effective in enhancing affinity towards both cytosolic isoforms, while retaining high (> 100‐fold) selectivity for Hsp90*β* (D'Amico et al. 2025). However, such groups do not provide the means to incorporate electrophilic warheads. **3a**, **3b**, and **3d** failed to bind Hsp90*α*, while their apparent *K*_*D*_ values for Hsp90*β* ranged from 3.82–5.24 *μ*M. These data agree with prior reports that highlight the influence of secondary amines on Hsp90*β* affinity/selectivity (Mishra, Liu, et al. 2021). Nevertheless, these intermediates' lack of affinity towards Hsp90*α* and ~20‐fold selectivity for Hsp90*β* support their use for the incorporation of electrophilic warheads.

**TABLE 1:** Apparent *K*_*D*_ values of Hsp90*β*‐selective inhibitor intermediates **3a**, **3b**, and **3d**, as determined via the “standard” FP assay.

Entry	Hsp90*α K*_*D*_ (*μ*M)	Hsp90*β K*_*D*_ (*μ*M)	Selectivity for Hsp90*β*
**1**	2.36 ± 0.09	0.023 ± 0.002	103‐fold
**3a**	> 100	4.78 ± 0.39	> 21‐fold
**3b**	> 100	3.82 ± 0.31	> 26‐fold
**3d**	> 100	5.24 ± 0.41	> 19‐fold

### 3.3 Time‐Dependent Assessment of Hsp90 Binding Affinity

It is important to remember that this “standard” FP assay captures the inhibitors' apparent binding affinities toward Hsp90 at a specific moment in time (i.e., after a ~24 h incubation). This is a necessary distinction, as the binding of TCIs is a two‐step process that begins with occupation of the ligand‐binding site, followed by covalent bond formation with the target residue. The rate at which this second step occurs is influenced by numerous factors, such as the inherent reactivity/electrophilicity of the TCI warhead. Therefore, a time‐dependent FP assay was utilized to characterize the binding of **4a–i** and **5** to cytosolic Hsp90s over a 24 h interval (Table 2).

**TABLE 2:** Apparent *K*_*D*_ values of Hsp90*β*‐selective covalent inhibitors against Hsp90*β*, as determined via the time‐dependent FP assay over a 24 h period.

	Entry	Time (h)					
		0.25	0.50	0.75	1	2	24
Hsp90*β K*_*D*_ (nM)	**1**	16.15	5.46	6.04	5.31	5.05	6.79
	**4a**	130.9	112.8	189.3	206.8	131	693.1
	**4b**	191.3	211.4	236.5	289.5	303.1	2593
	**4c**	925.2	1169	1994	2861	2947	> 10,000
	**4d**	85.3	21.8	29.4	31	18.3	55
	**4e**	74.6	37.8	27.1	20.7	18	43.6
	**4f**	243.3	155.3	199.5	160.3	155.7	1228
	**4g**	209.6	169.8	166.5	163.9	204.7	1094
	**4h**	213.9	219.2	158.1	110.2	164.5	883.8
	**4i**	1015	899.3	612.3	534.6	528.2	6506
	**5**	201.9	115.2	89.2	76.5	50.8	158.5

Over this time interval, compound **1**, the non‐covalent inhibitor used in these experiments as a control, manifested an IC_50_ value of 16.15 nM at 0.25 h, reached a minimum value of 5.05 nM at 2 h, which then increased to 6.79 nM at 24 h. For the derivatives with an ethylenediamine linker (**4a–c**), only **4a** behaved in a similar manner as **1**, with a relatively low apparent *K*_*D*_ = 131 nM at 2 h that increased to 693 nM at 24 h. Meanwhile, the apparent *K*_*D*_ values for both **4b** and **4c** started at much higher concentrations (191.3 nM and 925.2 nM, respectively at 0.25 h) and increased to low micromolar values over this same period. For the piperazine tether‐bearing analogs (**4d–f**), all three behaved similarly to **1** and manifested minimum IC_50_ values at 2 h. Interestingly, both **4d** and **4e** were among the most potent inhibitors tested, with IC_50_ values of ~18 nM at 2 h, which increased to ~50 nM after 24 h. The 4‐aminopiperidine‐containing inhibitors (**4 g–i**) were largely ineffective, since their activity was reminiscent to that of **1**, but with much higher apparent *K*_*D*_ values that also increased to the low micromolar values at 24 h. Lastly, fluorosulfonyl derivative **5** exhibited a similar profile as **4d** and **4e**, with a minimum apparent *K*_*D*_ value of 50.8 nM at 2 h that roughly tripled to 158.5 nM at 24 h. The data from these compounds provide valuable insights with regards to the optimal warhead(s) and tether length for engagement with Lys58. For instance, all three acrylamide warheads permit reactivity at the electrophilic *β*‐carbon, while the fluorophenyl acrylamide can also react at the 4‐position. However, derivatives with this latter warhead (**4c**, **4f**, and **4i**) exhibited the lowest affinity in the assay. Despite having two electrophilic sites, it's plausible that the phenyl ring of this moiety sterically blocks access to the *β*‐carbon, while the *para‐* position lies at a less‐than‐ideal distance/angle to allow engagement with Lys58. The other two acrylamides demonstrated comparable activities to each other, regardless of the linker (**4a** vs. **4b**, **4d** vs. **4e**, and **4 g** vs. **4 h**). Interestingly, the activities of **4d**, **4e**, and **5** (all of which feature the piperazine linker) most closely resembled that of **1**, both in terms of low nanomolar IC_50_ values, as well as how these values change over time. Consequently, it's reasonable to conclude that the ethylenediamine tether allows too much rotation for the warheads, while the 4‐aminopiperidine situates them too far from Lys58. Therefore, piperazine appears to possess the appropriate balance of distance and flexibility, as suggested by a prior study (D'Amico et al. 2025).

The inhibitors generally exhibited maximal binding to Hsp90*β* after 2 h, which led to their assessment against Hsp90*α* for the same amount of time (Table 3). Interestingly, **1** manifested ~2000‐fold selectivity for Hsp90*β* over Hsp90*α* after 2 h incubation, as compared to the 103‐fold selectivity observed after 24 h (apparent *K*_*D*_'s for these isoforms = 0.023 and 2.36 *μ*M, respectively). These data demonstrate a rapid binding of inhibitors to Hsp90*β*, followed by slower binding to Hsp90*α*. Similar trends were also noted for the covalent inhibitors, as most of these compounds displayed no affinity for Hsp90*α* at 10,000 nM. Consequently, high Hsp90*β* selectivity is best exemplified by **4d**, **4e**, and **5**, values for which appear to lie between 197‐ and ~550‐fold for the desired isoform.

**TABLE 3:** Apparent *K*_*D*_ values of Hsp90*β*‐selective covalent inhibitors, as determined via the time‐dependent FP assay at 2 h.

Entry	Hsp90*α K*_*D*_ (nM)	Hsp90*β K*_*D*_ (nM)	Selectivity for Hsp90*β*
**1**	> 10,000	5.05	> 1980
**4a**	> 10,000	131	> 76
**4b**	> 10,000	303.1	> 33
**4c**	> 10,000	2947	> 3
**4d**	< 10,000	18.3	< 546
**4e**	< 10,000	18	< 556
**4f**	> 10,000	155.7	> 64
**4 g**	> 10,000	204.7	> 49
**4 h**	> 10,000	164.5	> 61
**4i**	> 10,000	528.2	> 19
**5**	> 10,000	50.8	> 197

Although these data reveal the time‐dependent nature of the inhibitors' binding to Hsp90*β*, they do not indicate whether covalent bond formation occurs with Lys58. Consequently, incubation of Hsp90*β* with **4d** or **5** was followed by several mass spectrometry analyses to confirm the presence of Hsp90*β*‐inhibitor covalent adducts; unfortunately, the experiments were inconclusive as the corresponding mass was not detected.

### 3.4 In Vitro Evaluation of Hsp90*β*‐Selective Covalent Inhibitors

As previously discussed, the mechanism of TCIs is a time‐dependent process that can lead to sustained engagement with a target protein. Several recent studies have demonstrated the safety profile of Hsp90*β* inhibitors, as revealed by low antiproliferative activity against normal human retinal (ARPE‐19) and breast cells (MCF‐10A), as well as murine prostate cells and fibroblasts (L cells) (Rahmy et al. 2022; Reynolds, Mishra, and Blagg 2025; Reynolds, Hu, et al. 2025). These results are supplemented by in vivo safety/efficacy in the contexts of immunotherapy and opioid therapy, while a more extensive safety profile revealed the anticancer activity to be more reminiscent of pimitespib (approved in Japan for the treatment of gastrointestinal stromal tumors) than the Hsp90 *pan*‐inhibitor 17‐AAG (Rahmy et al. 2022; Reynolds, Mishra, and Blagg 2025; Duron et al. 2024). Therefore, the inhibitors were evaluated via an MTS cell viability assay against the A549, MDA‐MB‐231, and SKOV‐3 human cancer cell lines after a 72 h incubation (Table 4).

**TABLE 4:** Antiproliferative IC_50_ values of covalent Hsp90*β*‐selective inhibitors, as determined via an MTS cell viability assay.

Entry	IC_50_ (*μ*M)		
	A549	MDA‐MB‐231	SKOV‐3
**1**	0.604 ± 0.053	0.050 ± 0.002	0.185 ± 0.017
**4a**	5.35 ± 0.11	2.06 ± 0.12	3.28 ± 0.06
**4b**	7.08 ± 0.13	2.06 ± 0.03	3.73 ± 0.16
**4c**	7.34 ± 0.36	2.55 ± 0.09	7.11 ± 0.41
**4d**	2.19 ± 0.16	1.18 ± 0.03	2.13 ± 0.17
**4e**	3.59 ± 0.23	2.25 ± 0.02	3.08 ± 0.23
**4f**	3.19 ± 0.15	5.00 ± 0.22	13.92 ± 0.64
**4 g**	7.27 ± 0.32	4.84 ± 0.34	7.72 ± 0.06
**4 h**	7.86 ± 0.38	3.76 ± 0.19	3.59 ± 0.24
**4i**	9.95 ± 0.35	9.01 ± 0.70	36.03 ± 1.44
**5**	2.83 ± 0.17	3.14 ± 0.26	4.25 ± 0.13

As has been previously reported, compound **1** exhibited nanomolar activity against all three cell lines (D'Amico et al. 2025). Meanwhile, the covalent inhibitors **4a–i** and **5** generally manifested activity in the low micromolar range. Among the compounds evaluated in this study, **4d**, **4e**, and **5** displayed the highest affinities toward Hsp90*β*, with double‐digit nanomolar *K*_*D*_ values over 24 h. Consequently, these three analogues similarly displayed the most potent activity against these cell lines. Furthermore, the poor affinities of the fluorophenyl‐containing derivatives (**4c**, **4f**, and **4i**) resulted in diminished *in cellulo* activity. Intriguingly, the MDA‐MB‐231 cell line appears to be sensitive to Hsp90*β* inhibition, while the A549 and SKOV‐3 cells appear more resistant, as has been observed with other recently reported Hsp90*β*‐selective inhibitors (Serwetnyk et al. 2025; D'Amico et al. 2025; Reynolds, Hu, et al. 2025).

A potential explanation for this observation was recently reported by Mersich and coworkers, whose multi‐omics analyses revealed that, in resistant cell lines, Hsp90*β* inhibition can be overcome via compensatory Hsp90*α* expression and aryl hydrocarbon receptor activation to manifest metabolic rewiring, cytoskeletal remodeling, and other adaptive mechanisms (Mersich et al. 2025).

## 4 Conclusion

In conclusion, the preparation and characterization of proposed Hsp90*β*‐selective covalent inhibitors resulted in analogues that maintain high affinity and selectivity, as best represented by compounds **4d**, **4e**, and **5**. The data obtained from the derivatives reveal the ability of their electrophilic *α*,*β*‐unsaturated carbonyl or fluorosulfonyl warheads to engage with Hsp90*β* at nanomolar concentrations. However, our inability to isolate protein‐inhibitor covalent adducts suggests these compounds may operate via a mechanism of action that is independent of Lys58 modification. Nevertheless, insights regarding the kinetics of inhibitors binding the Hsp90 N‐terminal ATP‐binding pocket were obtained from this study. Interestingly, after a 2 h incubation, many of these compounds displayed the greatest affinity towards Hsp90*β*, while comparable binding was non‐existent against Hsp90*α*. Even so, the affinities exhibited by these electrophilic analogues were lower than that of the non‐covalent Hsp90*β*‐selective inhibitor, **1**, indicating that further optimization is necessary.

## Author Contributions

**Terin D'Amico:** formal analysis, investigation, writing – original draft. **Tyelor S. Reynolds:** formal analysis, investigation. **Michael A. Serwetnyk:** formal analysis, investigation, writing – original draft. **Brian S. J. Blagg:** conceptualization, writing – review and editing, supervision.

## Funding

This work was supported by grants awarded to B.S.J.B. from the National Institutes of Health (DA052340).

## Conflicts of Interest

The authors declare the following competing financial interest(s): B.S.J.B. is a co‐founder of Grannus Therapeutics, which aims to bring Hsp90*β*‐selective inhibitors toward clinical development. B.S.J.B., T.D., and M.A.S. are co‐inventors on a patent pending related to this work.

## Supporting information


**Data S1:** cbdd70290‐sup‐0001‐Supinfo.docx.

## Data Availability

The data that supports the findings of this study are available in the Supporting Information of this article.
