# Supplementary material for: Attempts to Achieve Targeted Covalent Inhibition of Hsp90β
Source: Chem Biol Drug Des. 2026 Apr 12;107(4):e70290. doi: 10.1111/cbdd.70290 (PMC13070582; doi:10.1111/cbdd.70290)
Supplement: Supplementary file 1 — Data S1: cbdd70290‐sup‐0001‐Supinfo.docx. [file CBDD-107-e70290-s001.docx]

**Supporting Information**

**Attempts to Achieve Targeted Covalent Inhibition of Hsp90*β***

Terin D’Amico, Tyelor S. Reynolds, Michael A. Serwetnyk, and Brian S. J. Blagg*

Department of Chemistry and Biochemistry, Warren Center for Drug Discovery, The University of Notre Dame, Notre Dame, Indiana 46556, United States of America

* = Corresponding author: [bblagg@nd.edu](mailto:bblagg@nd.edu)

**Table of Contents**

1. Characterization of Intermediates and Compounds – **S3–10**
2. NMR Spectra of All New Compounds – **S11–28**
3. Supplemental References – **S28**
4. **Characterization of Intermediates and Compounds**

*6-(3-ethyl-6,6-dimethyl-4-oxo-4,5,6,7-tetrahydro-1H-indazol-1-yl)-3-propyl-8-(tetrahydro-2H-pyran-4-yl)isoquinolin-1(2H)-one (****1****).* **Characterization of *1* is consistent with a prior report.^1^**

*6-(3-ethyl-6,6-dimethyl-4-oxo-4,5,6,7-tetrahydro-1H-indazol-1-yl)-8-fluoro-3-propylisoquinolin-1(2H)-one (****2****).* Yield 28%, 110 mg. **Characterization of *2* is consistent with a prior report.^2^**

*6-(3-ethyl-6,6-dimethyl-4-oxo-4,5,6,7-tetrahydro-1H-indazol-1-yl)-8-(methyl(2-(methylamino)ethyl)amino)-3-propylisoquinolin-1(2H)-one (****3a****).* Yield 70%, 408 mg; ^1^H NMR (400 MHz, Chloroform-*d*) δ 9.49 (s, 1H), 7.05–6.99 (m, 2H), 6.21 (s, 1H), 3.45 (t, *J* = 6.0 Hz, 2H), 2.97 (d, *J* = 9.2 Hz, 5H), 2.89–2.81 (m, 4H), 2.53 (t, *J* = 7.5 Hz, 2H), 2.44 (s, 3H), 2.41 (s, 2H), 1.76 (h, *J* = 7.4 Hz, 3H), 1.32 (t, *J* = 7.5 Hz, 3H), 1.10 (s, 6H), 1.02 (t, *J* = 7.3 Hz, 3H). ^13^C NMR (101 MHz, CDCl_3_) δ 193.17, 162.22, 155.72, 155.44, 149.30, 142.98, 142.39, 141.63, 116.66, 115.14, 111.76, 110.85, 104.24, 55.06, 52.53, 49.24, 42.37, 37.55, 36.51, 35.85, 34.96, 28.44 (2), 21.38, 21.24, 13.59, 12.94. HRMS (ESI/Q-TOF) *m/z*: [M + H] Calc’d for C_27_H_38_N_5_O_2_, 464.3020, found 464.3010.

*6-(3-ethyl-6,6-dimethyl-4-oxo-4,5,6,7-tetrahydro-1H-indazol-1-yl)-8-(piperazin-1-yl)-3-propylisoquinolin-1(2H)-one (****3b****).* Yield 68%, 400 mg; ^1^H NMR (400 MHz, DMSO-*d*_6_) δ 10.94 (s, 1H), 7.21 (d, *J* = 2.1 Hz, 1H), 6.94 (d, *J* = 2.1 Hz, 1H), 6.32 (s, 1H), 3.52 (s, 4H), 3.06–3.00 (m, 2H), 2.96 (dd, *J* = 9.1, 4.8 Hz, 5H), 2.83 (q, *J* = 7.5 Hz, 2H), 2.41 (t, *J* = 7.5 Hz, 2H), 2.34 (s, 2H), 1.63 (h, *J* = 7.4 Hz, 2H), 1.21 (t, *J* = 7.5 Hz, 3H), 1.01 (s, 6H), 0.91 (t, *J* = 7.3 Hz, 3H). ^13^C NMR (101 MHz, DMSO) δ 193.15, 161.33, 155.37, 154.46, 150.42, 144.17, 143.21, 141.47, 116.44, 115.20, 112.12, 108.90, 103.03, 53.68 (2), 52.34, 45.71 (2), 36.88, 35.83, 34.30, 28.23 (2), 21.59, 21.22, 13.91, 13.31. HRMS (ESI/Q-TOF) *m/z*: [M + H] Calc’d for C_27_H_36_N_5_O_2_, 462.2864, found 462.2866.

*tert-butyl (1-(6-(3-ethyl-6,6-dimethyl-4-oxo-4,5,6,7-tetrahydro-1H-indazol-1-yl)-1-oxo-3-propyl-1,2-dihydroisoquinolin-8-yl)piperidin-4-yl)carbamate (****3c****).* Yield 86%, 628 mg; ^1^H NMR (400 MHz, Chloroform-*d*) δ 12.19 (s, 1H), 7.82 (s, 1H), 7.02 (s, 2H), 6.20 (d, *J* = 12.5 Hz, 1H), 3.75 – 3.61 (m, 2H), 3.45 (d, *J* = 9.6 Hz, 1H), 2.96 (q, *J* = 7.5 Hz, 2H), 2.84 (s, 2H), 2.75 (d, *J* = 13.2 Hz, 2H), 2.52 (q, *J* = 8.1 Hz, 2H), 2.41 (s, 2H), 2.10 (d, *J* = 8.5 Hz, 3H), 1.74 (h, *J* = 7.5 Hz, 2H), 1.53 (s, 7H), 1.44 (d, *J* = 4.1 Hz, 3H), 1.31 (t, *J* = 7.5 Hz, 3H), 1.10 (s, 6H), 0.98 (t, *J* = 7.3 Hz, 3H). ^13^C NMR (101 MHz, CDCl_3_) δ 193.20, 163.01, 160.73, 157.55, 155.90, 155.62, 149.34, 144.29, 143.33, 141.33, 116.55, 112.90, 109.77, 103.85, 79.78, 53.07 (2), 52.54, 49.98, 37.49, 35.86, 34.85, 32.85, 31.68, 28.54 (2), 28.44 (2), 28.39, 22.03, 21.37, 13.73, 12.98. HRMS (ESI/Q-TOF) *m/z*: [M + H] Calc’d for C_33_H_46_N_5_O_4_, 576.3544, found 576.3534.

*8-(4-aminopiperidin-1-yl)-6-(3-ethyl-6,6-dimethyl-4-oxo-4,5,6,7-tetrahydro-1H-indazol-1-yl)-3-propylisoquinolin-1(2H)-one (****3d****).* Yield 77%, 400 mg; ^1^H NMR (400 MHz, Chloroform-*d*) δ 9.36 (s, 1H), 7.04 (d, *J* = 8.6 Hz, 2H), 6.22 (s, 1H), 3.54 (d, *J* = 11.0 Hz, 2H), 2.96 (q, *J* = 7.5 Hz, 3H), 2.84 (s, 4H), 2.56 (t, *J* = 7.6 Hz, 2H), 2.41 (s, 2H), 2.15–1.95 (m, 3H), 1.95–1.65 (m, 5H), 1.31 (t, *J* = 7.5 Hz, 3H), 1.11 (s, 6H), 1.00 (t, *J* = 7.3 Hz, 3H). ^13^C NMR (101 MHz, CDCl_3_) δ 193.13, 162.16, 155.81, 149.32, 143.04, 141.83, 116.74 (2), 112.64, 110.24, 104.30, 52.49, 37.63, 35.86, 34.79, 28.46 (2), 21.37, 21.29, 13.57, 12.95. HRMS (ESI/Q-TOF) *m/z*: [M + H] Calc’d for C_28_H_38_N_5_O_2_, 476.3020, found 476.3007.

*N-(2-((6-(3-ethyl-6,6-dimethyl-4-oxo-4,5,6,7-tetrahydro-1H-indazol-1-yl)-1-oxo-3-propyl-1,2-dihydroisoquinolin-8-yl)(methyl)amino)ethyl)-N-methylacrylamide (****4a****).* Yield 22%, 25 mg; ^1^H NMR (400 MHz, Chloroform-*d*) δ 9.83 (s, 1H), 7.16 – 6.85 (m, 2H), 6.68 – 5.98 (m, 3H), 5.51 (ddd, *J* = 21.9, 10.4, 2.1 Hz, 1H), 3.73 (d, *J* = 9.2 Hz, 2H), 3.63 – 3.37 (m, 1H), 3.29 (d, *J* = 7.4 Hz, 1H), 3.19 – 2.99 (m, 2H), 2.99 – 2.76 (m, 8H), 2.51 (q, *J* = 10.7, 7.7 Hz, 2H), 2.36 (s, 2H), 1.71 (h, *J* = 6.6 Hz, 2H), 1.25 (td, *J* = 7.5, 1.6 Hz, 3H), 1.05 (d, *J* = 2.0 Hz, 6H), 0.96 (td, *J* = 7.3, 3.1 Hz, 3H). ^13^C NMR (126 MHz, CDCl_3_) δ 193.43 and 193.31 (rotamers), 166.80 and 166.66 (rotamers), 162.85 and 162.80 (rotamers), 156.09 and 155.93 (rotamers), 155.19 and 154.86 (rotamers), 149.58, 143.38, 143.09 and 142.73 (rotamers), 142.01, 128.16 and 128.00 (rotamers), 127.76 and 127.70 (rotamers), 117.05 and 116.96 (rotamers), 115.13 and 114.34 (rotamers), 112.69 and 112.00 (rotamers), 110.85 and 110.18 (rotamers), 104.82 and 104.76 (rotamers), 56.63 and 54.21 (rotamers), 52.69, 48.01, and 45.76 (rotamers), 41.62 and 41.33 (rotamers), 37.87 and 37.74 (rotamers), 36.06 and 36.04 (rotamers), 35.99 and 35.10 (rotamers), 34.62, 28.63 (2), 21.55 and 21.52 (rotamers), 21.47, 13.82 and 13.81 (rotamers), 13.18 and 13.10 (rotamers). HRMS (ESI/Q-TOF) *m/z*: [M + H] Calc’d for C_30_H_40_N_5_O_3_, 518.3126, found 518.3126.

*(E)-N-(2-((6-(3-ethyl-6,6-dimethyl-4-oxo-4,5,6,7-tetrahydro-1H-indazol-1-yl)-1-oxo-3-propyl-1,2-dihydroisoquinolin-8-yl)(methyl)amino)ethyl)-N-methylbut-2-enamide (****4b****).* Yield 29%, 33 mg; ^1^H NMR (400 MHz, Chloroform-*d*) δ 9.89 (d, *J* = 75.6 Hz, 1H), 7.19 – 6.91 (m, 2H), 6.79 (ddq, *J* = 39.4, 14.0, 6.8 Hz, 1H), 6.32 – 6.00 (m, 2H), 3.82 – 3.66 (m, 2H), 3.51 (s, 1H), 3.37 (t, *J* = 7.8 Hz, 1H), 3.13 (d, *J* = 20.5 Hz, 2H), 3.03 (d, *J* = 6.4 Hz, 1H), 3.01 – 2.81 (m, 7H), 2.55 (t, *J* = 7.7 Hz, 2H), 2.41 (s, 2H), 1.96 – 1.69 (m, 5H), 1.31 (td, *J* = 7.5, 2.4 Hz, 3H), 1.10 (d, *J* = 4.8 Hz, 6H), 1.02 (td, *J* = 7.3, 3.3 Hz, 3H). ^13^C NMR (101 MHz, CDCl_3_) δ 193.24 and 193.11 (rotamers), 166.73, 162.45, 155.87 and 155.73 (rotamers), 154.91, 149.43 and 149.36 (rotamers), 143.18 and 142.89 (rotamers), 141.78 and 141.59 (rotamers), 132.06 and 131.31 (rotamers), 121.62 and 121.55 (rotamers), 117.73 and 117.60 (rotamers), 116.83, 114.96, 112.42, 110.59, 104.50 and 104.42 (rotamers), 56.29, 52.48, 47.62, 45.38 and 41.24 (rotamers), 37.67 and 37.55 (rotamers), 35.84, 35.74 and 35.62 (rotamers), 34.93 and 34.42 (rotamers), 28.44 (2), 21.37, 21.31, 18.18, 13.66 and 13.63 (rotamers), 12.96 and 12.91 (rotamers). HRMS (ESI/Q-TOF) *m/z*: [M + H] Calc’d for C_31_H_42_N_5_O_3_, 532.3282, found 532.3271.

*(E)-N-(2-((6-(3-ethyl-6,6-dimethyl-4-oxo-4,5,6,7-tetrahydro-1H-indazol-1-yl)-1-oxo-3-propyl-1,2-dihydroisoquinolin-8-yl)(methyl)amino)ethyl)-3-(4-fluorophenyl)-N-methylacrylamide (****4c****).* Yield 5%, 7 mg; ^1^H NMR (400 MHz, Chloroform-*d*) δ 9.01 (d, *J* = 177.5 Hz, 1H), 7.62 – 7.37 (m, 3H), 7.16 – 6.97 (m, 4H), 6.64 – 6.16 (m, 2H), 3.98 (t, *J* = 7.7 Hz, 1H), 3.85 (q, *J* = 6.6, 5.9 Hz, 1H), 3.50 (dt, *J* = 15.6, 6.8 Hz, 1H), 3.30 (dd, *J* = 9.0, 6.3 Hz, 1H), 3.13 – 3.01 (m, 5H), 3.01 – 2.90 (m, 2H), 2.90 – 2.81 (m, 3H), 2.51 (t, *J* = 7.6 Hz, 1H), 2.46 – 2.34 (m, 3H), 1.74 (q, *J* = 7.5 Hz, 1H), 1.61 (p, *J* = 7.4 Hz, 1H), 1.33 – 1.24 (m, 3H), 1.10 (d, *J* = 2.0 Hz, 6H), 0.99 (dt, *J* = 15.0, 7.3 Hz, 3H). ^13^C NMR (126 MHz, CDCl_3_) δ 193.27, 166.92, 164.48 (d, *J* = 551.1 Hz), 162.15, 156.10 and 155.89 (rotamers), 155.42 and 155.00 (rotamers), 149.53, 143.31 and 143.25 (rotamers), 142.59 and 142.34 (rotamers), 142.05, 141.44 and 141.25 (rotamers), 130.06 and 130.00 (rotamers), 129.76 (2, d, *J* = 8.4 Hz), 117.96, 117.29, 116.08 (d, *J* = 12.9 Hz), 115.91 (2, d, *J* = 12.9 Hz), 112.73 and 111.93 (rotamers), 110.72 and 110.11 (rotamers), 104.58 and 104.49 (rotamers), 57.49, 54.37, 52.68, 48.16 and 46.12 (rotamers), 41.68 and 40.64 (rotamers), 37.88 and 37.80 (rotamers), 36.16 and 36.04 (rotamers), 35.17 and 35.02 (rotamers), 28.63 (2), 21.56 and 21.53 (rotamers), 21.39, 13.79 and 13.76 (rotamers), 13.08 and 13.03 (rotamers). ^19^F NMR (376 MHz, CDCl_3_) δ -110.45, -110.57, -110.83, -111.00, -111.86, -112.16, -112.33, -112.56. HRMS (ESI/Q-TOF) *m/z*: [M + H] Calc’d for C_36_H_43_FN_5_O_3_, 612.3304, found 612.3343.

*8-(4-acryloylpiperazin-1-yl)-6-(3-ethyl-6,6-dimethyl-4-oxo-4,5,6,7-tetrahydro-1H-indazol-1-yl)-3-propylisoquinolin-1(2H)-one (****4d****).* Yield 28%, 31 mg; ^1^H NMR (400 MHz, Chloroform-*d*) δ 9.96 (s, 1H), 7.12 (d, *J* = 2.0 Hz, 1H), 7.04 (d, *J* = 2.1 Hz, 1H), 6.63 (dd, *J* = 16.8, 10.5 Hz, 1H), 6.33 (dd, *J* = 16.8, 2.0 Hz, 1H), 6.27 (s, 1H), 5.73 (dd, *J* = 10.5, 2.0 Hz, 1H), 3.87 (s, 3H), 3.22 (s, 4H), 2.96 (q, *J* = 7.5 Hz, 2H), 2.85 (s, 2H), 2.57 (t, *J* = 7.6 Hz, 2H), 2.42 (s, 2H), 1.75 (h, *J* = 7.5 Hz, 3H), 1.31 (t, *J* = 7.5 Hz, 3H), 1.11 (s, 6H), 1.00 (t, *J* = 7.3 Hz, 3H). ^13^C NMR (126 MHz, CDCl_3_) δ 193.25, 165.72, 162.53, 156.16, 155.16, 149.50, 143.39, 143.14, 142.22, 128.25, 127.68, 117.11, 115.80, 113.56, 110.44, 104.58, 53.78, 52.75, 52.67, 46.34, 42.45, 37.91, 36.08, 35.11, 28.67 (2), 21.56, 21.42, 13.73, 13.09. HRMS (ESI/Q-TOF) *m/z*: [M + H] Calc’d for C_30_H_38_N_5_O_3_, 516.2969, found 516.2962.

*(E)-8-(4-(but-2-enoyl)piperazin-1-yl)-6-(3-ethyl-6,6-dimethyl-4-oxo-4,5,6,7-tetrahydro-1H-indazol-1-yl)-3-propylisoquinolin-1(2H)-one (****4e****).* Yield 43%, 49 mg; ^1^H NMR (400 MHz, Chloroform-*d*) δ 9.15 (s, 1H), 7.12 (d, *J* = 2.0 Hz, 1H), 7.02 (d, *J* = 2.1 Hz, 1H), 6.97 – 6.86 (m, 1H), 6.32 (dq, *J* = 14.9, 1.6 Hz, 1H), 6.25 (d, *J* = 2.0 Hz, 1H), 3.86 (s, 4H), 3.41–3.04 (m, 4H), 2.97 (q, *J* = 7.5 Hz, 2H), 2.85 (s, 2H), 2.54 (t, *J* = 7.5 Hz, 2H), 2.42 (s, 2H), 1.90 (dd, *J* = 6.9, 1.6 Hz, 3H), 1.74 (h, *J* = 7.4 Hz, 2H), 1.31 (t, *J* = 7.5 Hz, 3H), 1.12 (s, 6H), 1.01 (t, *J* = 7.3 Hz, 3H). ^13^C NMR (126 MHz, CDCl_3_) δ 193.26, 165.90, 162.42, 156.15, 155.23, 149.49, 143.37, 143.04, 142.22, 142.05, 121.65, 117.11, 115.79, 113.51, 110.40, 104.57, 53.79, 52.85, 52.68, 46.22, 42.37, 37.91, 36.08, 35.12, 28.67 (2), 21.56, 21.40, 18.51, 13.72, 13.09. (ESI/Q-TOF) *m/z*: [M + H] Calc’d for C_31_H_40_N_5_O_3_, 530.3126, found 530.3127.

*(E)-6-(3-ethyl-6,6-dimethyl-4-oxo-4,5,6,7-tetrahydro-1H-indazol-1-yl)-8-(4-(3-(4-fluorophenyl)acryloyl)piperazin-1-yl)-3-propylisoquinolin-1(2H)-one (****4f****).* Yield 14%, 18 mg; ^1^H NMR (400 MHz, Chloroform-*d*) δ 10.00 (s, 1H), 7.71 (d, *J* = 15.4 Hz, 1H), 7.62–7.50 (m, 2H), 7.24–7.13 (m, 2H), 7.09 (t, *J* = 8.6 Hz, 2H), 6.91 (d, *J* = 15.4 Hz, 1H), 6.33 (s, 1H), 4.03 (s, 3H), 3.32 (s, 4H), 2.99 (q, *J* = 7.5 Hz, 3H), 2.88 (d, *J* = 11.3 Hz, 2H), 2.62 (t, *J* = 7.5 Hz, 2H), 2.45 (s, 2H), 1.78 (h, *J* = 7.4 Hz, 2H), 1.33 (t, *J* = 7.5 Hz, 3H), 1.14 (s, 6H), 1.03 (t, *J* = 7.3 Hz, 3H). ^13^C NMR (126 MHz, CDCl_3_) δ 193.26, 165.62, 163.74 (d, *J* = 250.1 Hz), 162.18, 156.18, 155.16, 149.50, 143.35, 142.90, 142.26, 141.94, 131.70, 129.79 (2, d, *J* = 8.4 Hz), 117.13, 117.01–116.99 (m), 116.15 (2, d, *J* = 21.8 Hz), 115.81, 113.55, 110.46, 104.56, 53.85, 52.83, 52.67, 46.39, 42.67, 37.92, 36.09, 35.18, 28.68 (2), 21.56, 21.40, 13.74, 13.09. ^19^F NMR (376 MHz, CDCl_3_) δ -110.58, -112.05. HRMS (ESI/Q-TOF) *m/z*: [M + H] Calc’d for C_36_H_41_FN_5_O_3_, 610.3188, found 610.3173.

*N-(1-(6-(3-ethyl-6,6-dimethyl-4-oxo-4,5,6,7-tetrahydro-1H-indazol-1-yl)-1-oxo-3-propyl-1,2-dihydroisoquinolin-8-yl)piperidin-4-yl)acrylamide (****4g****).* Yield 26%, 26 mg; ^1^H NMR (400 MHz, Chloroform-*d*) δ 9.07 (s, 1H), 7.06 (dd, *J* = 12.9, 2.0 Hz, 2H), 6.30 (dd, *J* = 16.9, 1.5 Hz, 1H), 6.23 (s, 1H), 6.11 (dd, *J* = 16.9, 10.2 Hz, 1H), 5.71 (d, *J* = 8.0 Hz, 1H), 5.65 (dd, *J* = 10.2, 1.5 Hz, 1H), 4.10 (s, 1H), 3.59 – 3.46 (m, 2H), 2.96 (p, *J* = 9.7, 8.6 Hz, 3H), 2.85 (s, 2H), 2.52 (t, *J* = 7.5 Hz, 2H), 2.42 (s, 2H), 2.13 (dd, *J* = 12.7, 4.0 Hz, 2H), 1.89 (dtd, *J* = 23.6, 10.4, 9.8, 5.5 Hz, 2H), 1.72 (h, *J* = 7.4 Hz, 3H), 1.31 (t, *J* = 7.5 Hz, 3H), 1.11 (s, 6H), 1.00 (t, *J* = 7.3 Hz, 3H). ^13^C NMR (126 MHz, CDCl_3_) δ 193.30, 165.08, 162.22, 156.09, 155.82, 149.52, 143.22, 142.55, 142.14, 131.26, 126.66, 117.01, 115.74, 113.07, 110.53, 104.55, 52.69, 52.46 (2), 46.51, 37.87, 36.07, 35.13, 32.47 (2), 28.66 (2), 21.57, 21.39, 13.76, 13.14. HRMS (ESI/Q-TOF) *m/z*: [M + H] Calc’d for C_31_H_40_N_5_O_3_, 530.3126, found 530.3120.

*(E)-N-(1-(6-(3-ethyl-6,6-dimethyl-4-oxo-4,5,6,7-tetrahydro-1H-indazol-1-yl)-1-oxo-3-propyl-1,2-dihydroisoquinolin-8-yl)piperidin-4-yl)but-2-enamide (****4h****).* Yield 8%, 8 mg; ^1^H NMR (400 MHz, Chloroform-*d*) δ 8.67 (s, 1H), 7.18 – 6.99 (m, 2H), 6.98 – 6.80 (m, 1H), 6.23 (s, 1H), 5.89 – 5.75 (m, 1H), 4.07 (s, 1H), 3.52 (s, 2H), 3.06 – 2.88 (m, 4H), 2.85 (s, 2H), 2.50 (t, *J* = 7.6 Hz, 2H), 2.42 (s, 2H), 2.18 – 2.04 (m, 2H), 1.87 (td, *J* = 7.5, 6.8, 1.7 Hz, 4H), 1.72 (h, *J* = 7.6 Hz, 4H), 1.32 (td, *J* = 7.5, 1.3 Hz, 3H), 1.11 (s, 6H), 1.01 (t, *J* = 7.4 Hz, 3H). ^13^C NMR (126 MHz, CDCl_3_) δ 193.10, 165.30, 162.18, 155.90, 149.32, 143.02, 141.97, 139.95, 138.51, 131.46, 125.23, 120.01, 116.82, 114.64, 110.30, 104.55, 52.49, 52.36 (2), 41.83 (2), 37.67, 35.86, 34.94, 32.36, 28.46 (2), 21.36, 21.24, 17.72, 13.54, 12.92. HRMS (ESI/Q-TOF) *m/z*: [M + H] Calc’d for C_32_H_42_N_5_O_3_, 544.3242, found 544.3276.

*(E)-N-(1-(6-(3-ethyl-6,6-dimethyl-4-oxo-4,5,6,7-tetrahydro-1H-indazol-1-yl)-1-oxo-3-propyl-1,2-dihydroisoquinolin-8-yl)piperidin-4-yl)-3-(4-fluorophenyl)acrylamide (****4i****).* Yield 11%, 15 mg; ^1^H NMR (400 MHz, Chloroform-*d*) δ 8.87 (s, 1H), 7.60 (d, *J* = 15.5 Hz, 1H), 7.55 – 7.41 (m, 2H), 7.14 – 6.96 (m, 4H), 6.33 (d, *J* = 15.6 Hz, 1H), 6.23 (s, 1H), 5.81 (s, 1H), 4.14 (s, 1H), 3.61 – 3.49 (m, 2H), 2.97 (q, *J* = 7.5 Hz, 3H), 2.85 (s, 2H), 2.52 (t, *J* = 7.6 Hz, 2H), 2.42 (s, 2H), 2.24 – 2.12 (m, 2H), 1.95 (qd, *J* = 11.6, 10.9, 3.6 Hz, 2H), 1.72 (dd, *J* = 14.9, 7.4 Hz, 3H), 1.32 (t, *J* = 7.5 Hz, 3H), 1.11 (s, 6H), 1.01 (t, *J* = 7.3 Hz, 3H). ^13^C NMR (126 MHz, CDCl_3_) δ 193.29, 165.28, 163.73 (d, *J* = 250.2 Hz), 162.11, 156.10, 155.88, 149.51, 143.22, 142.47, 142.18, 140.04, 131.31 (d, *J* = 3.4 Hz), 129.78 (2,d, *J* = 8.3 Hz), 120.82 (d, *J* = 1.5 Hz), 117.03, 116.13 (2,d, *J* = 22.0 Hz), 115.79, 113.07, 110.55, 104.54, 52.70, 52.46 (2), 46.81 (2), 37.88, 36.07, 35.15, 32.56, 28.66 (2), 21.57, 21.38, 13.75, 13.13. ^19^F NMR (376 MHz, CDCl_3_) δ -105.60, -109.40, -110.10, -110.27, -110.50, -110.52, -110.72, -111.36, -111.46, -111.78, -111.79, -111.95. HRMS (ESI/Q-TOF) *m/z*: [M + H] Calc’d for C_37_H_43_FN_5_O_3_, 624.3344, found 624.3345. **NOTE:** A small amount of ethyl acetate was added to help solubilize this compound in CDCl_3_. Consequently, peaks in the ^1^H and ^13^C NMR spectra that correspond to this co-solvent are indicated as such.

*4-(6-(3-ethyl-6,6-dimethyl-4-oxo-4,5,6,7-tetrahydro-1H-indazol-1-yl)-1-oxo-3-propyl-1,2-dihydroisoquinolin-8-yl)piperazine-1-sulfonyl fluoride (****5****).* Yield 20%, 12 mg; ^1^H NMR (400 MHz, Chloroform-*d*) δ 8.67 (s, 1H), 7.11 (dd, *J* = 12.4, 2.0 Hz, 2H), 6.26 (d, *J* = 1.9 Hz, 1H), 3.78 (s, 4H), 3.32 (s, 4H), 2.97 (q, *J* = 7.5 Hz, 2H), 2.86 (s, 2H), 2.53 (t, *J* = 7.5 Hz, 2H), 2.43 (s, 2H), 1.74 (h, *J* = 7.4 Hz, 2H), 1.32 (t, *J* = 7.5 Hz, 3H), 1.13 (s, 6H), 1.02 (t, *J* = 7.3 Hz, 3H). ^13^C NMR (101 MHz, CDCl_3_) δ 193.03, 161.53, 156.10, 154.46, 149.31, 143.09, 142.64, 142.10, 117.00, 115.61, 113.65, 110.53, 104.36, 52.45 (2), 51.79, 47.38 (2), 37.73, 35.90, 34.99, 28.47 (2), 21.36, 21.15, 13.50, 12.91. ^19^F NMR (471 MHz, CDCl_3_) δ 37.04. HRMS (ESI/Q-TOF) *m/z*: [M + H] Calc’d for C_27_H_35_FN_5_O_4_S, 544.2388, found 544.2401.

1. **NMR Spectra of New Intermediates and Compounds**
   1. ^1^H and ^13^C NMR Spectra for **3a**

- 1. ^1^H and ^13^C NMR Spectra for **3b**

- 1. ^1^H and ^13^C NMR Spectra for **3c**

- 1. ^1^H and ^13^C NMR Spectra for **3d**

- 1. ^1^H and ^13^C NMR Spectra for **4a**

- 1. ^1^H and ^13^C NMR Spectra for **4b**

- 1. ^1^H, ^13^C, and ^19^F NMR Spectra for **4c**

- 1. ^1^H and ^13^C NMR Spectra for **4d**

- 1. ^1^H and ^13^C NMR Spectra for **4e**

- 1. ^1^H, ^13^C, and ^19^F NMR Spectra for **4f**

- 1. ^1^H and ^13^C NMR Spectra for **4g**

- 1. ^1^H and ^13^C NMR Spectra for **4h**

- 1. ^1^H, ^13^C, and ^19^F NMR Spectra for **4i**

- 1. ^1^H, ^13^C, and ^19^F NMR Spectra for **5**

1. **Supplemental References**
2. D’Amico, T.; Serwetnyk, M. A.; Dou, X.; Mersich, I.; Barlow, D.; Houseknecht, K. L.; Streicher, J. M.; Ali, A.; Blagg, B. S. J. Hsp90*β*-Selective Inhibitors: Probing the Solvent-Accessible Frontier. *ChemMedChem* **2025**, *20* (24), e202500657. DOI: <https://doi.org/10.1002/cmdc.202500657>
3. Serwetnyk, M. A.; Strunden, T.; Mersich, I.; Barlow, D.; D’Amico, T.; Mishra, S. J.; Houseknecht, K. L.; Streicher, J. S.; Blagg, B. S. J. Optimization of an Hsp90*β*-selective inhibitor *via* exploration of the ATP-binding pocket. *Eur. J. Med. Chem.* **2025**, *297*, 117925. DOI: <https://doi.org/10.1016/j.ejmech.2025.117925>
